# Integrating Primary Care Into Community Mental Health Centres in Texas, USA: Results of a Case Study Investigation

**DOI:** 10.5334/ijic.4630

**Published:** 2019-10-29

**Authors:** Rebecca Wells, Ellen D. Breckenridge, Sasha Ajaz, Aman Narayan, Daniel Brossart, James H. Zahniser, Jolene Rasmussen

**Affiliations:** 1 The University of Texas School of Public Health, Department of Management, Policy, and Community Health, Houston, TX, US; 2 UT Southwestern Medical School, Dallas, TX, US; 3 Counseling Psychology Program, Department of Educational Psychology, Texas A&M University, TX, US; 4 Meadows Mental Health Policy Institute, Dallas, TX, US; 5 Adult Behavioral Health, Texas Council of Community Centers, Austin, TX, US

**Keywords:** integration, reverse integration, colocation, mental health, primary care

## Abstract

**Introduction:**

Despite evidence that people with serious mental illness benefit from receiving primary care within mental health care settings, there is little research on this type of integration. The objective of this study was to characterize how providers and patients experienced implementation of primary care into specialty mental health services.

**Methods:**

During site visits, study team members interviewed staff and conducted focus groups with patients at 10 United States community mental health centres then beginning to integrate primary into their practices. One year later, follow up phone interviews with key centre staff informants validated and updated findings. Data analysis included thematic coding of results from staff interviews and patient focus groups.

**Results:**

Findings included the importance of the scope of primary care services provided on site, given limited alternatives available to patients; rapid scale-up; overcoming challenges in provider recruitment and retention; and adaptations to engage patients as well as to improve communication between mental health and primary care providers.

**Conclusion:**

Providers and patients perceived improvements through integrated care. However, the majority of patients were uninsured, and the funding was short term. The long-term viability of integrated care for community mental health centre patients may hinge on adequate, predictable public funding.

## Introduction

Perhaps no group of individuals would benefit more from enhanced primary care access than would people with serious mental illnesses, as they have much higher rates than the general population of chronic diseases [1,2], poor diet, tobacco use [3,4], and inadequate primary care use [5,6,7], as well as shortened life expectancy [2,8,9].

Although individuals with serious mental illness may receive psychiatric care, they often have limited primary care [5,6,7,10]. Even when people with serious mental illness access primary care, results are often suboptimal, partly because physicians may misattribute physical illness to mental illness [11] or because these patients often need more time than do other patients to communicate with their providers [12]. Hence, expert consensus supports integrating physical health care services into mental health care settings for people with serious mental illness [13,14].

There is now substantial research on integration of mental health care into primary care [15]. However, research is limited on how mental health providers integrate primary care into their practices [16]. Such studies have shown integration of physical care into mental health care for individuals with serious mental illness to be associated with increased preventive care [17,18,19,20] and improved health outcomes [17,19,21]. Studies also tend to find that patients with serious mental illness receiving integrated care use hospitals less than do patients receiving usual care [17,18,19,20].

Prior research on integration of physical into mental health care has identified barriers including space constraints [18]; difficulty recruiting and retaining primary care providers and patients [17,22], as well as incorporating primary care providers into integrated teams [23]; and limited sharing of medical records between primary care providers and mental health providers [16,24]. Other challenges have included problems with billing [24] and aggregate data collection and reporting [17,24]. However, the generality of such findings has been unknown, because these studies have focused on initiatives with required components [17,22] or been limited to a few facilities [18,23]. The purpose of the current study was to extend understanding of how to integrate primary care into mental health care for people with serious mental illness by identifying how 10 independent community mental health centres across one state have done this for their patients.

## Methods: Study context and data collection

The study was designed through a collaboration among Texas A&M University; the University of Texas; the Texas Health and Human Services Commission, which funds community health centres in that state; the Meadows Mental Health Policy Institute, a non-profit organisation devoted to improving mental health in Texas; and the Texas Council of Community Centres, which is the membership organization for community mental health centres in that state. Because of limited prior research on primary care integration into mental health care and an interest in processes within local contexts, a mixed methods comparative case study was identified as the optimal empirical approach, drawing primarily on staff interviews and patient focus groups [25].

In 2014, the study partners chose to examine ten of the 33 community mental health centres in Texas that were using Medicaid 1115 waiver funding to integrate primary care into their existing behavioural health services for patients with serious mental illness. Medicaid is the primary health insurance program for people in the United States with low incomes. Although the federal government specifies what services Medicaid covers and how providers are paid, states may secure waivers allowing alternative program structures intended to improve health care quality and cost effectiveness. Through Section 1115 waivers, many states have experimented with paying for innovative services based on attainment of performance measures. In Texas, the 1115 waiver includes a value-based pool through which providers can earn payments for a range of initiatives. Many Medicaid waiver initiatives have addressed behavioural health care, including integration of mental health care into physical health care, as well as the focus of the current study, integration of physical health care into mental health care.

Ten community mental health centres were selected for the current study to represent potentially promising practices in every major region in Texas, as well as diversity in rurality [26], poverty rates, race, and ethnicity [27,28,29,30]. The Texas Council of Community Centers approached community mental health centre executive directors about participating; all agreed. The research protocol was approved by the Texas A&M and University of Texas IRBs. All study participants received information about the study and their rights and provided written informed consent.

Data sources included interviews with community mental health centre agency leadership, front line clinical and administrative staff, and focus groups with patients receiving integrated care. Staff interviews and the first eight patient focus groups were conducted during site visits between October 2014 and January 2015. All sites were initiating integrated care during these visits, eight of which were serving patients at that time. In May 2015, members of the research team re-visited a ninth site that had begun serving patients at that point, to conduct a final focus group. Two researchers conducted the first two site visits together, and each then led additional site visits with another member of the team assisting in each visit. Interviews and focus groups were conducted in English and audio recorded when acceptable to participants, professionally transcribed, and reviewed by a member of the research team for accuracy. On a few occasions, a team member instead took detailed notes. One year later, key community mental health centre staff informants were interviewed by phone to verify initial characterizations of their sites and provide updates on their programs.

During each site visit, the key informant was asked about the project’s origins as well as prospects for sustainability. Questions in interviews with additional professionals at sites addressed the nature of integration, based on the TriWest Group’s Person-Cantered Healthcare Home Fidelity Scales [31,32] and dimensions identified through prior research as potentially affecting integration implementation, which were dichotomized as present or absent for the current study (e.g., remodelled existing clinic space, yes or no). These dimensions included adaptability to specific community mental health centre contexts, perceived complexity, community mental health centre and partner organizational structure and culture, formal and informal communication quality, goals and feedback, and provider and patient engagement [17,22,23,33].

Patient focus group recruitment materials emphasized the voluntary nature of participation and lack of consequences for choosing not to participate. Patients who participated in these focus groups received a gift card and lunch. Patients were asked which services they were receiving through integrated care, what had been helpful about those services, and what could be improved. To allow each patient in each focus group an opportunity to answer each question, a round robin process was used, continuing until everyone indicated that they had no new responses for that question. A second member of the research team wrote down each response on a numbered list, projected on a wall or written on a large piece of paper so that everyone could see. Sometimes participants would confirm a prior response rather than providing a new response, and sometimes the moderator would ask if a response could be combined with a prior related response (e.g., access to two different types of medication becoming combined into ‘access to medication’). This yielded a list of patient-generated themes relating to the nature of integration services they received, the perceived benefits of those services, and areas of potential improvement, as well as comments relating to these themes.

## Analysis

Analysis began with a summary of integration at each community mental health centre, including a profile of the context, structure, and history to date of integration at that centre [31]. These initial within-case analyses yielded classification of integration structure into community mental health centres that hired primary care providers versus those that partnered with independent primary care providers as the research team identified a pattern of differences between these two integration structures in, for instance, ease of acculturating primary care providers.

Interview comments on the distinctive structures of federally qualified health centres, such as enhanced billing rates for primary care and federal reporting requirements, led to division of the latter category into community mental health centre partnerships with federally qualified health centres versus with other types of primary care providers. United States federally qualified health centres receive federal grants and additional enhanced reimbursement rates in exchange for serving all patients in medically underserved areas or populations regardless of ability to pay, and submitting annual reports to the Department of Health and Human Services. Collectively, approximately 1,400 federally qualified health centres serve almost 10% of the United States population [34].

Three members of the research team thematically coded interview and focus group transcripts, notes, and field memos in ATLAS.ti version 7.5.17 (Berlin, Scientific Software Development, 2015). A constant comparison approach was used [30], beginning with factors identified from prior research [17,22,23] and employing extensive analytic memoing. Codes were refined through team meetings, during which discrepancies were discussed until consensus was achieved [30]. For example, initially separates codes for comprehensiveness and ease of information sharing, respectively, were merged when the two dynamics emerged as closely intertwined, and a new code for patient education emerged from frequent references to this focus within patient encounters.

A member of the research team combined patient focus group responses across sites, with input from the principal investigator. For instance, patients in five focus groups had identified medication-related benefits of integration. In order to focus on common patient experiences, only responses that had emerged in at least three of the nine focus groups were retained. Members of the research team then reviewed transcripts and notes from the focus groups for patient quotes related to each such theme.

## Results

Sixty-six staff were interviewed across the 10 community mental health centres during the October 2014–January 2015 site visits. Staff participants’ roles included community mental health centre-level administration, supervising integrated services, front line clinical care, and coordination. Twelve of the staff in the study were physicians, four were nurse practitioners, and five were licensed clinical social workers. The average organizational tenure was seven years. Between one and four key staff informants from each site participated in follow up calls with members of the study team one year later; these calls focused on verifying initial characterizations of integration and providing select updates, such as related to staff turnover during the time since the initial site visits.

Seventy-five adult patients at the nine sites that were operational between October 2014 and May 2015 participated in focus groups. Consistent with community mental health centre patient records (not shown), more than 60% of focus group participants were uninsured, and almost all reported annual incomes under $15,000/year (Table 1).

**Table 1:** Attributes of Patients Who Participated in Focus Groups (N = 75 across 9 sites).

Characteristic	N	(%)

Age (N = 74),years, mean ± SD	49 ± 9	
Male	42	56
Female	33	44
Race/ethnicity (N = 75)		
White	34	45
Black	15	20
Hispanic	29	39
Other	3	4
Education (N = 75)		
No general education development (GED)/equivalent	11	15
GED or high school diploma	38	51
Some college	22	29
College degree or higher	4	5
Mental health diagnoses (N = 75)		
Bipolar	28	37
Schizophrenia	18	24
Depression	58	77
Other	24	32
Primary care diagnoses (N = 75)		
Hypertension	46	61
Diabetes	29	39
COPD (chronic obstructive pulmonary disease)	15	20
Asthma	9	12
Other	27	36
Insurance status (N = 74)		
Uninsured	46	61
Insured		
Medicaid	12	16
Medicare	5	7
Dual eligible (Medicaid + Medicare)	7	9
Other insured	5	7
Income (N = 71)		
0–$14,999	69	97
$15,000–$34,999	2	3
Living situation		
Live alone (N = 74)	26	35
Homeless within last year (N = 72)	31	41
Reported reliable access to transportation (N = 73)	54	74

Numbers may >100% because participants could select multiple responses to the question or because of rounding.

## Description of the care practices

Of the 10 community mental health centres, four hired primary care providers, four contracted with federally qualified health centres for primary care at the community mental health centres, and two contracted with independent primary care providers (Table 2). These differing configurations had implications for information sharing, provider acculturation, and billing.

**Table 2:** Attributes of Integration across Community Mental Health Centers (N = 10).

	CMHC only (N = 4)		CMHC + FQHC (N = 4)		CMHC + other PC (N = 2)		Overall (N = 10)	

Integration attributes	N	%	N	%	N	%	N	%

Physical facilities								
Remodeled existing clinic space	4	100	4	100	2	100	10	100
Had usual PC physical exam rooms	4	100	4	100	2	100	10	100
PC and MH on same floor of same building	2	50	3	75	2	100	7	70
On-site pharmacy	2	50	0	0	0	0	2	20
On-site lab sample collection	3	75	4	100	1	50	8	80
On-site dental practice	0	0	1	25	0	0	1	10
Scope of practice								
Health education								
Nutrition	4	100	3	75	1	50	8	80
Exercise	4	100	3	75	0	0	7	70
Used a specific exercise coaching model	0	0	1	25	0	0	1	10
Smoking cessation coaching	4	100	3	75	2	100	9	90
Used specific smoking cessation model	2	50	2	50	1	50	5	50
Tangible rewards for progress	1	25	1	25	1	50	3	30
Staff recruitment and retention								
PCP employed by CMHC	4	100	1	25	1	50	6	60
Loss of PCP delayed/paused primary care	2	50	1	25	1	50	4	40
Other primary care turnover in first year	1	25	1	25	0	0	2	20
CMHC/partner staff members providing integrated care, mean ± SD, range								
# FTEs in first year of operations	7 ± 2	4–9	12 ± 9	4–27	6 ± 3	4–9	9 ± 6	4–27
# FTEs in second year of operations	14 ± 13	4–37	13 ± 8	7–26	7 ± 3	3–10	12 ± 10	3–37
% growth in integrated team size between 1st and 2nd interview (1 year apart)		106		6		11		39
% FTEs departed between 1st and 2nd interview		36		37		57		41
Patient engagement								
Warm hand-offs (MHC → PC)	2	50	4	100	2	100	8	80
Walk-in physical care available	3	75	4	100	2	100	9	90
Total # patients in first year of operations	2,717		2,934		157		5,808	
Total # patients in second year of operations, % increase from first year	3,747	38	6,101	108	793	405	10,641	83
Information sharing								
Electronic health records	4	100	1	25	2	100	7	70
Common health records	4	100	3	75	1	50	8	80
Behavioral health care liaison or care coordinator	3	75	4	100	2	100	9	90
Review performance data	1	25	3	75	0	0	4	40
Use clinical pathways	1	25	1	25	0	0	2	20

Abbreviations: CMHC, community mental health center; FQHC, Federally Qualified Health Center; MHC, mental health care provider; PC, primary care; PCP, primary care professional.

### Physical facilities

Most community mental health centres renovated rather than built new facilities for integrated care; one used a mobile van to reach multiple service areas (Table 2). Sometimes space constraints necessitated separate check-in areas or splitting physical and mental health services across floors.

Two community mental health centres provided prescriptions through on-site pharmacies; some others had prescriptions mailed to patients. One community mental health centre provided on-site dental services, which were described by both patients and providers as much-needed.

“My medication kills my teeth. I need dental care.” Patient, site 1

“Because most all of our patients do not have dental care … When you look at how dental care affects health and heart disease, it’s huge.” Psychiatrist, site 7 (which did not provide dental care)

### Patient care flow

Initial referrals into primary care began during mental health visits at the community mental health centres. When mental health providers identified a physical health need, they asked whether the patient had an existing primary care provider. Patients who did not have access to regular primary care (or, in some instances, were unwilling to return to a primary care provider) were shown back to a waiting room. Those who were new to integrated care completed an intake process specific to physical health care, including determining eligibility for sliding scale fees. Often primary care copayments were higher than those for mental health care, which confused patients, and therefore took time to explain. Depending on primary care provider availability and patient needs and preferences, patients either made appointments to return for primary care, typically within a week, or saw the primary care provider on the day of the referral. The primary care visit itself was like a typical such visit in a self-standing context, except that it was embedded in mental health treatment processes. For instance, when primary care staff saw emergent mental health needs, they contacted mental health professionals on site, who then also assessed the patients during the encounter.

“Everybody else does mental health into primary care … I said let’s do it the other way. Let’s bring them in to us.” Administrator, site 7 (community mental health centre that hired primary care providers)

“We’ve built out a primary care suite, so we’ve got six exam rooms, an exam table—it looks like a little primary care clinic. We’ve built that out within our outpatient mental health clinic, but [name omitted] is the federally qualified health centre that we’re working with. They inhabit that clinic. It’s kind of theirs.” Administrator, site 10

### Scope of practice

The scope of primary care practice was important because patients often had limited access to alternative health care. Two community mental health centres narrowed their scope of practice over time, in one instance as the primary care provider found patients had unexpectedly common pain and sleep problems. Another community mental health centre expanded practice beyond the initial “top 10” conditions such as diabetes and hypertension. Consistencies in integrated care included coaching in smoking cessation, exercise, and nutrition (Table 2).

“A patient complains of chest pain and she needs a stress test, and she’s unfunded, and I can’t do anything for her. I try to optimize her medication, try to keep her on all medicines she’s supposed to be on, give her a nitro pill. If the pain gets bad, go to the emergency room.” Primary care physician, site 2 (community mental health centre–federally qualified health centre partnership)

“Then there’s a couple of clinics locally that’ll charge $40.00 a visit. They have to pay in cash. Unfortunately for some of our clients that’s not reasonable.” Administrator, site 4 (community mental health centre that hired primary care providers)

“I haven’t followed up with all of it [dermatology, mammogram, colonoscopy, inhaler] because [the local] Clinic doesn’t offer it.” Patient, site 3 (community mental health centre that hired primary care providers)

“He calls, and he’ll ask questions, ‘I’m struggling with this. I’m struggling with that,’ and so I always use positive reinforcement. Always, even if he feels like he’s gotten off the wagon and drank too many sodas. Then we’ll say, ‘Okay, but tomorrow you can have unsweetened tea, and you can use the Sweet ‘n’ Low or drink more water and exercise.’” Navigator, site 5 (community mental health centre–federally qualified health centre partnership), on coaching for a patient with psycho-affective disorder, alcohol dependence, and diabetes

## Integration implementation dynamics

### Staff recruitment and retention

Four community mental health centres delayed or paused operations after losing a primary care provider and two community mental health centres reported loss of a primary care provider shortly after their hiring. Overall, six of the 10 community mental health centres experienced non-operational periods because of lack of primary care providers; this affected community mental health centres with both internal and external primary care providers. One reason for primary care provider retention challenges was differences between physical and mental health care cultures. For instance, some staff reported higher general stress and greater pressure to meet patient volume goals in mental than standard physical health care. Problematic cultural differences appeared to be less common when community mental health centres directly hired primary care staff. Overall staff turnover was high, although integrated care teams were also growing during the study period.

“The biggest challenge first came from hiring the physician.” Administrator, site 4 (community mental health centre that hired primary care providers)

“Part of the difficulty is finding primary care and then someone who’ll take an interest in this population … because I am very concerned about your clinical ability, but I’m also much more concerned about, do you have a heart for this population of patients? … That speaks to how difficult it has been to find someone.” Administrator, site 10 (community mental health centre–federally qualified health centre partnership)

### Patient engagement

Staff at some sites described low initial patient volume as allowing time for staff and patients to adjust to integrated care. For instance, staff sometimes reported simplifying patient health education or learning to take more time to communicate with patients. Some sites also enhanced integrated care entry processes, including simplifying written referrals or calling patients on the phone about appointments. Other ways of engaging patients included frequently escorting them from mental health to physical health spaces, which integrated staff referred to as “warm hand-offs,” offering same-day and walk-in appointments, and providing transportation to community mental health centres. Evidence of success for these strategies as a whole included the average number of patients served almost doubling in one year (Table 2).

“We started examining the number of people we referred that actually penetrated into primary care. We were like, ‘Ah, it’s not good enough.’ We took it down and said, ‘Hey, you have to actually warm handoff them. You can’t leave. If nobody’s there, you wait until somebody shows up and gives them an appointment.’” Administrator, site 2 (community mental health centre-federally qualified health centre partnership)

In focus groups, the most commonly cited benefits of integrated care were medication-related services, identified in five of the nine sessions, and caring staff, mentioned in six of the nine sessions. The most common response to the prompt about how integration could be improved was greater provider availability, mentioned in three focus groups. Patients in five focus groups reported improving their diet in response to integrated care. Patients in all nine groups attributed a range of physical and mental health improvements to integrated care. Comments included observations that integrated care alleviated their anxiety about physical conditions, reduced physical symptoms, and helped with living fuller, more positive lives.

“My medical doctor listened to me and is open to me when I am having difficulties with medications.” Patient, site 5 (community mental health centre-federally qualified health centre partnership)

“The providers here care, and they listen to patients.” Patient, site 6 (community mental health centre-non federally qualified health centre partnership)

“I’m more aware of what I’m eating.” Patient, site 1 (community mental health centre-federally qualified health centre partnership)

“When you don’t know what’s going on with your body it’s scary. Just being able to get the information about my medical needs has been great.” Patient, site 8 (community mental health centre that hired primary care providers)

### Information sharing

Improving information sharing between physical and mental health staff was a pervasive quest. By the second year of the study, most sites had electronic health records (which may or may not have been shared between physical and mental health care), a single integrated health record (which may or may not have been electronic), and staff responsible for coordinating physical and mental health care. Integrated records yielded immediate access to information about all prescribed medications. Both staff working with and without integrated health records developed work-arounds to ensure that each discipline had adequate information when meeting with patients. Staff at sites without shared records sometimes printed record extracts. Two sites modified information systems to enable mental health and primary care providers to see each other’s entries more readily and, in one instance, to sign off on a single treatment plan.

“The intention of this communication [within the electronic health record] is for it to be in the chart, for it to be actual medical record. We don’t want it to be some sort of instruction or question that needs to be answered by the other practitioner. We don’t want it to be that, because then it opens for liability, all those concerns. It’s not what our intention is, so we’re trying to make it as an FYI type of box comment that is still in the chart, that is important, that is there, but that the other provider can read, consider, ignore or address if they feel it’s necessary.” Psychiatrist, site 6 (community mental health centre-non federally qualified health centre partnership)

Almost half the sites used aggregated outcomes data to track performance. Few had formalized clinical pathways providing guidance to clinicians for managing comorbid conditions, which take time to develop or adapt to new settings.

“We had to really work on that process of getting the labs, the meds, and all that. That’s been some fine-tuning.” Administrator, site 2 (community mental health centre-federally qualified health centre partnership)

“I think people leave [integrated team case conferences] and they’re like—if I’m a case manager and I say, ‘Wow, I didn’t know that the patient is going through that.’ Or vice versa, the psychiatrist, ‘Wow, I didn’t know that [had] that impact.’ Everybody leaves with much more understanding of how to better care for that one individual. It’s been very beneficial.” Primary care provider, site 4 (which hired primary care providers)

### Sustainability

Key informants at four community mental health centres reported previous unsuccessful attempts at integrating primary care into their practices, generally attributed to resource constraints. One common initial obstacle to integration was an inability to bill Medicaid or Medicare (the public health insurance covering people ages 65 and older) because the new contracts needed in the context of the Medicaid waiver had not yet been approved. Another major challenge was integrating billing systems. However, given more than half of patients having no insurance, the potential of billing strategies to cover the costs of integration was inherently limited.

“Maybe assisting individuals to apply for Medicaid, or help them navigate the process so we can get more individuals involved in Medicaid. We can bill for those services and then sustain the model more effectively.” Administrator, site 6 (community mental health centre-non federally qualified health centre partnership)

“We’re in the process of getting all of the Medicare and Medicaid payments for the [primary care provider] viable in our offices. They’re so picky! You’d think that the change in scope [allowed through the Medicaid waiver] would allow for that, but it’s a whole different process. We’re trying to get all that to happen.” Administrator, site 5 (community mental health centre-federally qualified health centre partnership)

“[The plan is] try to document enough results that then we can go back to the MCOs [managed care organizations, which pay for services primarily on a per-patient rather than per encounter basis] and say, ‘Look, if y’all contribute this much, or raise rates by this much, we can keep this project going, which will reduce your costs over here in these other areas.’” Administrator, site 7 (community mental health centre that hired primary care providers)

“We’re looking into grants and all kinds of other revenues, other insurance. We’re going to have to do something.” Chief operating officer, site 9 (community mental health centre-non federally qualified health centre site)

Of the nine centres the evaluation team was able to reach for follow up in the winter of 2019, all but one was still providing integrated care. One of the responding centres reported being unable to sustain integrated care, due to having very few patients with health insurance, and Medicaid waiver funding shifting to other priorities. This centre has continued physical screening and referrals to physical health care, although there are few such resources for individuals without insurance. Another centre also cited a low percentage of insured patients and changing Medicaid waiver funding as reasons for possible future cessation of integrated care. Among the other seven centres, four had expanded integrated care, two of which cited additional funding from the United States Substance Abuse and Mental Health Services Administration (SAMHSA) grants for Promoting Integration of Primary and Behavioral Health Care (PIPBHC).

## Discussion

Given emerging evidence of integration’s benefits for people with serious mental illness [17,18,19,20,21], this study sought to characterize integration in a state-wide United States sample of community mental health centres. Findings suggest the importance of the range of services provided on-site, given limited alternatives; success scaling up integration quickly, despite challenges in provider and patient recruitment and retention; adaptation in patient engagement and inter-professional communication strategies; and good coordination between physical and mental health providers, despite differences in professional culture and patient-related information. The sustainability of integrated services beyond the Medicaid waiver is uncertain. Benefits from patients’ perspectives included reduced stress and improved health.

This study’s design created some limitations. Community mental health centres were chosen for this study based on their potentially promising practices, which may limit the generality of findings to mental health centres with less implementation capacity. Texas’s low health care spending [35] and high rates of uninsurance [36] may also limit the generality of study findings to other countries or regions. However, the challenge of addressing complex health needs with limited resources is globally applicable [37,38,39]. The diversity and independence of community mental health centres in this study, as well as congruence of findings with those from prior research, suggest that both the challenges and potential benefits identified are likely to be broadly applicable.

Findings from this study have a number of implications for specialty mental health providers and policy makers seeking to integrate primary care into mental health care. For instance, on-site services assume particular importance for community mental health centre patients, who often have financial and transportation barriers to accessing health care [22]. On-site pharmacies appeared to improve convenience for patients, and may increase medication adherence as well as help clinicians track adherence. When that is not feasible, mail ordering prescriptions may be helpful for some patients.

The fact that only one community mental health centre was able to provide dental care on site raises a major opportunity for future improvement. Individuals with serious mental illness often have dental problems because of smoking, eating, and oral hygiene habits, as well as psychotropic medications that erode teeth [7]. In addition to pain, poor dental health has been linked with cardiovascular disease [40]. Participants at both the one site that offered dental services and sites that did not considered this a critical part of patient care. In general, scope of practice emerged as an important factor, given limited access to other providers. This suggests the potential value of supporting the full range of primary care within integrated community mental health centres, as well as related specialty care. When it is not feasible for community mental health centres to provide health care services, patients will benefit from any facilitation of access, including formal and informal referral agreements with external providers.

Community mental health centres in the current study increased their integrated care team size rapidly. However, this study also suggests that having low initial volume in integrated care can allow time for staff and patients to adjust to new processes that increase patient understanding and acceptance of integrated care.

As in prior research [17,22], the current study found that referrals alone often did not get community mental health centre patients into primary care, even within the same building. The frequency of warm hand-offs in the current sample, especially by the second year of integration, implies that administrators planning integrated care should allow staff time to physically escort patients between mental and physical health care. Other adaptations also tended to focus on connecting more directly with patients to engage them in integrated care.

Despite well-documented challenges with differing professional cultures and patient care information available [24], primary care and mental health providers in the current study reported positive coordination. Community mental health centres that hired primary care providers may be best positioned to share information because they can add primary care functionality to their existing patient records, and all providers have immediate access to the same information. Community mental health centres working with independent primary care providers may benefit from up-front investment in reducing legal and logistical barriers to sharing information. Community mental health centres partnering with federally qualified health centres appear to be the most constrained in this regard, as federally qualified health centre information systems must use federal reporting formats and standards.

Community mental health centres in the study had limited capacity for aggregate data tracking, as has been found in prior settings [16,24]. Funders might partner with community mental health centres to develop information systems, beginning with limited data pulls and analyses. These community mental health centres might then document for other centres how to develop such capacities. However, at least in Texas, community mental health centres’ only funding for data reporting comes through the Medicaid waiver. Hence, building information system capacity hinges on new funding sources for this purpose.

The current study yielded new evidence about how gaps in primary care provider availability can interrupt patient care [17,22], creating periods when services were not available. Research has indicated that a sense of belonging is important to professionals in integrated programs [24]. Investments in recruiting primary care providers who want to serve people with serious mental illness appear to be warranted, as well as acculturating them to mental health care. This may be particularly necessary when the primary care providers remain employees of other organizations, and so are less fully immersed in the mental health care environment. The frequent gaps in initial operations due to absence of primary care providers, combined with delays in insurance contracts, as well as benefits of time for providers and patients to adjust to integrated care, suggest allowing a cushion of time for implementation.

Frequent failed attempts at integration prior to the Medicaid waiver among centres in this study illustrates challenges sustaining these services in the absence of long-term funding. Federal and state health and human service agencies might support integration by expediting contract approval. Payers may also increase integration survival rates through providing adequate and bundled payments, including covering time spent on coordination. However, given the high proportion of community mental health centre patients who are uninsured, other dedicated revenue appears essential to sustaining integrated services.

Since this study was conducted, community mental health centres have continued preparing for value-based payment. Such efforts have included improving centre capacity for demonstrating cost and quality outcomes, through an initiative led by the Texas Council of Community Centers, with funding from the Robert Wood Johnson Foundation. A federal Certified Community Behavioral Health Clinic demonstration program also enables some centres to receive enhanced Medicaid reimbursement [41]. Federally qualified health centres, which have been at the forefront of integrated care for underserved populations, are key partners in these efforts. The biggest challenge Texas centres face in sustaining integrated care is the impending end of the state’s Medicaid 1115 waiver in 2022. At that point, the Texas Health and Human Services Commission hopes to continue funding many waiver innovations through Medicaid managed care. The Commission and Texas Council are also exploring ways to improve integrated care through a Medicaid program for people with disabilities called STAR+PLUS. However, community mental health centres will still be disadvantaged by virtue of serving a largely uninsured population.

The current study also complemented prior quantitative evidence of integration benefits [17,18,19,20,21] through patients’ perspectives on their experiences. The comfort patients feel with their existing mental health providers appears to be a major ingredient in integrated care engagement and success. Health education was a key part of integrated care in the current sample, reflecting the importance of lifestyle in managing chronic disease. Patient success in improving nutrition, physical activity, and sense of well-being may sustain engagement in integrated care as well as improve its outcomes.

## Conclusion

Over 50 years ago, President Kennedy sought federal funding for community mental health centres to “return mental health care to the main stream of American medicine” [42]. Findings from the current study indicate that specialty mental health care providers can integrate care for their clients, thereby improving routine care [17,18,19,20] and health outcomes [17,19,21]. Sustaining such programs appears dependent on recruiting and retaining primary care providers and engaging patients, all of whom face substantial adjustments transitioning from traditionally distinct physical and mental health care. The operational and clinical successes of integrated care, however, will not ensure its continuation. In the United States, many community mental health centres are funding integrated care in part through time-limited federal and state funding. In the long term, integrated care viability will depend on securing adequate, predictable funding [24].
